# FACTORS ASSOCIATED WITH SEXUAL SATISFACTION AMONG OLDER GAY MEN

**DOI:** 10.1093/geroni/igae098.2865

**Published:** 2024-12-31

**Authors:** Lucas Prieto, Deirdre Shires, Yuan Xiong

**Affiliations:** George Mason University, Fairfax, Virginia, United States; Michigan State University East Lansing, East Lansing, Michigan, United States; Michigan State University East Lansing, East Lansing, Michigan, United States

## Abstract

Internalization of ageist stereotypes or messages that consider the context of aging gay men is known as internalized gay ageism. Sexual satisfaction may be influenced by internalized gay ageism among aging gay men. This study aimed to explore internalized gay ageism and the potential relationship with sexual satisfaction and determine if body image was a mediator. A cross-sectional online survey collected data on sexual satisfaction and other variables related to sexual health and well-being among older gay men between December 2021 and May 2022. Participants were included in the study if they were 50 years or older, identified as gay, identified as male, assigned male at birth, and resided in the Midwestern United States. Analyses included descriptive, bivariate, and mediation. A total of 181 participants were recruited in the study, with a mean age of 65.29 years (SD=9.32). Overall, participants reported experiencing a low level of internalized gay ageism, positive body image, and a high level of sexual satisfaction. A complete mediation effect was found between internalized gay ageism and sexual satisfaction when mediated by body image [total effect b=-4.47, p=.001; direct effect b=.34, p=.80; indirect effect b=-4.82, 95% CI BootLL =-6.93, BootUL= -3.03]. Older gay men who were in open relationships were more sexually satisfied than older gay men who were single/widowed. Future research should continue to explore internalized gay ageism, relationship status, body image, and sexual satisfaction among older gay men.

